# Effectiveness of Omeprazole in Acid Peptic Disease: A Real-World, Patient-Reported Outcome Measures Study

**DOI:** 10.7759/cureus.41994

**Published:** 2023-07-17

**Authors:** Suresh Jain, Sandeep Shamrao Kulkarni, Jyoti R Mahapatra, Dilip Todi, Anup U Petare, Ritwik Banerjee, Rahul Rathod, Syed Naqvi, Amey Mane, Gauri Dhanaki, Bhavesh P Kotak

**Affiliations:** 1 Department of Gastroenterology, Digestive Endoscopy Clinic, Pune, IND; 2 Department of Gastroenterology, Sahyadri Super Speciality Hospital, Pune, IND; 3 Department of Gastroenterology, Peerless Hospital, Kolkata, IND; 4 Department of Gastroenterology, Narayana Superspeciality Hospital, Kolkata, IND; 5 Department of Medical Affairs, Dr. Reddy’s Laboratories Ltd., Hyderabad, IND; 6 Department of Medical Affairs, Dr. Reddy's Laboratories Ltd., Hyderabad, IND; 7 Department of Clinical Research, Dr. Reddy's Laboratories Ltd., Hyderabad, IND

**Keywords:** tsqm-9, pagi-sym, ppi, reflux, acid peptic disorder, omeprazole

## Abstract

Objectives

This prospective study assessed the effectiveness and patient satisfaction of four-week omeprazole therapy in acid peptic disease (APD).

Methods

This was an observational, post-marketing, real-world evidence, patient-reported outcome (PRO) measures study. Patients visiting the five study sites across India with symptoms of APD, and who were prescribed oral omeprazole (20/40 mg per day) for at least four weeks were enrolled after obtaining informed consent. Study assessments included frequency and severity of symptoms and overall satisfaction reported by the patients using the Patient Assessment of Gastrointestinal Disorder Symptom Severity Index (PAGI-SYM) questionnaire. The satisfaction with therapy was reported by the patients using the Treatment Satisfaction Questionnaire for Medication (TSQM) questionnaire. Both PAGI-SYM and TSQM were reported by patients on days 14 and 28. Omeprazole safety was assessed based on the adverse events reported by the patients.

Results

A total of 96 (62 males and 34 females) patients were included in the study, of which 38.54% had significant findings related to APD at baseline. The proportion of patients with symptoms reduced to 16.67% on day 14 and 8.33% on day 28 with omeprazole therapy. The PAGI-SYM total scores at baseline were 41.32 (15.487), which reduced to 20.86 (11.620) on day 14 (p < 0.0001), and to 8.93 (8.361) on day 28 (p < 0.0001). Significant reductions were also seen in individual symptom scores. The TSQM total scores increased to 36.67 (range: 13 to 63) on day 28 from 34.69 (range: 12 to 58) on day 14. Improvement in scores for all domains of TSQM (effectiveness, convenience, and global satisfaction) was seen on day 28. Improvement in reflux symptoms was reported by 46.74% and 68.48% of patients on day 14 and day 28, respectively. Four (4.17%) patients reported adverse events, which were of mild severity and were unrelated to omeprazole.

Conclusions

Omeprazole provides significant improvement in PAGI-SYM and TSQM questionnaires on day 14 and day 28. Patients reported the omeprazole-based therapy as effective, convenient, and satisfactory. Omeprazole therapy is safe and effective for the treatment of APD and shows good improvement in APD in patients suffering from duodenal ulcers, gastric ulcers, and reflux oesophagitis.

## Introduction

Acid peptic disease (APD) is a collective term used to include many conditions such as gastroesophageal reflux disease (GERD), gastritis, gastric ulcer, duodenal ulcer, esophageal ulcer, Zollinger-Ellison syndrome (ZES), and Meckel’s diverticular ulcer [1]. APD is caused by stress, smoking, alcohol, tobacco, or the use of corticosteroids and other drugs like non-steroidal anti-inflammatory drugs (NSAIDs), warfarin, niacin, some chemotherapy drugs, and spironolactone [2]. Other causes include a low-fiber diet, caffeinated drinks, or fatty foods in the diet, and many patients have a genetic predisposition. Excessive secretion of acid and pepsin or a weakened stomach mucosal defense is often responsible for damage to the mucosa resulting in ulceration [3]. Suppression of gastric acid secretion remains the main goal of therapy in patients with APD [4]. Proton pump inhibitors (PPIs) are the preferred treatments for the treatment of APD. Omeprazole is one of the first PPIs developed and used for reducing gastric acid secretion. PPIs suppress the basal and stimulated gastric acid secretion by inhibition of proton pump (H+/K+ ATPase) in the gastric parietal cells. PPIs help heal acid damage to the stomach and esophagus, help prevent ulcers, and may help prevent malignancy of the esophagus. Although many PPIs are developed after omeprazole like esomeprazole, pantoprazole, rabeprazole, lansoprazole, and dexlansoprazole, the use of omeprazole is still widespread due to its efficacy. The efficacy of omeprazole in APD is well documented, and in clinical studies, the newer, more potent PPIs, used at comparable doses have not shown greater efficacy than omeprazole [5-7]. However, real-world evidence studies of patients themselves reporting the effectiveness of omeprazole in improving the symptoms of APD are not common.

## Materials and methods

Setting

This practice-based, observational, post-marketing, multi-center, real-world evidence, patient-reported outcome (PRO) measures study was conducted at five tertiary care centers across India. The study sites were gastroenterology specialty clinics. The study protocol and the documents were approved by Narayana Superspeciality Hospital Ethics Committee (NSH-EC/AP/2020/007; dated: December 28, 2022).

Study participants

A total of 96 English-literate patients (62 male, 34 female) aged 18 to 65 years, diagnosed with APD were included in the study after obtaining written informed consent. Those who were prescribed capsule omeprazole monotherapy (20/40 mg per day) as per market authorization were included in the study. The prescriptions were at the discretion of the treating clinician. Patients with known hypersensitivity to any PPI, including omeprazole in the past, and those who participated in an investigational drug or investigational device study within 30 days prior to the start of the study were excluded. Prior treatment for APD like histamine-2 (H2) receptor antagonists or over-the-counter (OTC) antacids during the week immediately before the study enrolment was an exclusion. Patients with peptic ulcers who received anti-*Helicobacter pylori* drug regimens were excluded. Patients with any elective surgical procedure during the study duration or any medical condition according to the investigator, which may interfere with the treatment, were also not included in the study.

Study procedures

All data were collected and recorded in a case record form (CRF) to ensure strict confidentiality for any personal information. Patient information about their clinical condition on admission, comorbidities, complications, and details of other treatments received was collected. There were no study-specific treatments and all treatments for the patients were at the discretion of the treating clinician. Data of patients who completed at least four weeks of therapy with omeprazole 20/40 mg per day were included in the final analysis.

Study outcomes

The primary outcome was the symptom improvement score for APD assessed using the Patient Assessment of Gastrointestinal Disorder Symptom Severity Index (PAGI-SYM) questionnaire on day 14 [8]. Secondary outcomes were the symptom improvement score for APD assessed using the PAGI-SYM questionnaire on day 28, and the patient’s overall treatment satisfaction assessed using the Treatment Satisfaction Questionnaire for Medication (TSQM) on days 14 and 28 [9]. Other outcomes included the number of patients reporting improvement in reflux after the start of therapy, and clinical safety based on clinical adverse events (AEs) reported by the patients during the study. The AEs of special interest were gastrointestinal intolerance (abdominal pain, diarrhea, nausea, vomiting, heartburn), giddiness, and regurgitation.

Patient Assessment of Gastrointestinal Disorder Symptom Severity Index (PAGI-SYM)

The severity of gastrointestinal symptoms was assessed using the PAGI-SYM tool (English), which is a brief self-administered questionnaire (by the patient) consisting of 20 items. The items involved asking the patients to rate their gastrointestinal symptoms over the past 14 days using a six-point Likert scale, where 0 = no symptoms and 5 = very severe symptoms. The 20 items are clustered into six domains (nausea/vomiting, post-prandial fullness, bloating, upper abdominal pain, lower abdominal pain, and heartburn). The tool was used for the study since it has a short recall period (two weeks), which avoids recall bias. The PAGI-SYM assessments were done by patients at baseline and subsequently after treatment with omeprazole on day 14 and day 28.

Treatment Satisfaction Questionnaire for Medication (TSQM)

Treatment satisfaction was assessed using a brief questionnaire that will ask patients to rate their satisfaction with omeprazole treatment on day 14 and day 28. The TSQM tool (English) collects information about the symptoms (day-time heartburn, night-time heartburn, acid reflux, difficulty swallowing, and abdominal pain) present during the past 14 days. The TSQM has a total of 10 items that collect treatment satisfaction for three domains (effectiveness, convenience, and global satisfaction). Each item is rated by patients on a 1-10 scale. The highest score is 100 and a higher score is suggestive of greater treatment satisfaction. The TSQM assessments were done by patients after treatment with omeprazole on day 14 and day 28.

Clinical assessments

All patients underwent clinical assessments, medical history for any comorbid conditions, and concomitant medications used by the patients. Compliance was assessed based on patient recall for medication consumption, and consumption of >80% of prescribed medication was termed as compliant. All other conditions, comorbidities, and concomitant medications were monitored and recorded during the 28-day study period. No laboratory investigations were planned for the study purpose.

Statistical methods

The sample size was not based on any calculations and assumptions, and it was planned to collect data from about 400 patients from 10 sites across India. However, due to the SARS-CoV-2 pandemic, we were able to collect data from only 96 patients during the period from November 2020 to January 2022.

All data were entered into Microsoft Office Excel spreadsheet (Office version 365, Microsoft Corporation, Redmond, WA), and checked for errors and discrepancies. Data analysis was done using Windows-based Stata version 13 (StataCorp LLC, College Station, TX). Parametric data are presented as means with standard deviation (SD), whereas nominal and discrete data are presented as numbers with percentages. Data for the PAGI-SYM and TSQM scales are computed as total scores for respective domains and presented as means with SD. Change from baseline in the PAGI-SYM scores (domain and total) on days 14 and 28 were computed as paired differences and presented as means with SD along with 95% confidence intervals (CI) for the change. Data are presented as descriptives for different parameters. Pairwise comparisons of scores (baseline versus day 14 and baseline versus day 28) are analyzed for differences using the Wilcoxon test (non-parametric), whereas the Friedman test is used for the comparison of baseline scores with day 14 and day 28 scores for the total scores of PAGI-SYM. Scores for TSQM (patient satisfaction) are presented as descriptive for the different domains and total scores. All analyses were done using two-sided tests with alpha 0.05 (95% confidence levels).

## Results

Demography and baseline data

Data from all 96 patients (62 male, 34 female) were collected and evaluated as a per-protocol (PP) dataset for both efficacy and safety analyses.

The mean (SD) age of the patients was 39.28 (12.34) years with a body mass index (BMI) of 25.09 (3.39) kg/sq.m. Eighty-four (87.5%) patients received other medications, of which 58 (60.4%) received a single drug along with omeprazole, 26 (27.1%) received two drugs, 13 (13.5%) received three drugs, and five (5.2%) received four drugs. These were oral hypoglycemic agents, insulin, antihypertensive agents, laxatives, digestive enzymes, statins, benzodiazepines, antiplatelet, and benzodiazepines.

Table 1 presents the gender distribution and profile of patients in the study. Other comorbid conditions included gastrointestinal conditions other than APD (n = 4), and one patient each with anxiety/depression, epilepsy, hypercholesterolemia, and tuberculosis of the spine. Surgical history included appendectomy (n = 5), two patients each for inguinal hernia repair and laparotomy, and one patient each for surgery for fractures, hemorrhoidectomy, hysterectomy, and urinary tract surgery.

**Table 1:** Patient profile at baseline (n = 96) N/A: not available; WHO: World Health Organization.

		Total (n = 96)	
		No.	%
Gender	Male	62	64.58%
	Female	34	35.42%
Diet	Vegetarian	16	16.67%
	Non-vegetarian	14	14.58%
	Mixed diet	63	65.63%
	N/A	3	3.13%
Comorbidity	Diabetes mellitus	6	6.25%
	Hypertension	14	14.58%
	Heart disease	1	1.04%
	Thyroid disorders	1	1.04%
	Other disorders	8	8.33%
BMI category (WHO)	Underweight	1	1.04%
	Normal weight	44	45.83%
	Overweight	40	41.67%
	Obese	7	7.29%
	N/A	4	4.17%
Presenting symptoms	Heartburn	76	79.17%
	Abdominal pain	69	71.88%
	Vomiting	38	39.58%
	Nausea	66	68.75%
	Giddiness	8	8.33%
	Reflux	74	77.08%

PAGI-SYM scores

The reduction in total PAGI-SYM scores was by 20.46 (95% CI = 17.65 to 23.27) on day 14. Similarly, there were significant reductions in the individual symptom scores on day 28 (p < 0.0001). The total PAGI-SYM scores at baseline were 41.32 (15.487), which reduced to 8.93 (8.361) on day 28 (p < 0.0001). The reduction in total PAGI-SYM scores was by 32.40 (95% CI = 29.32 to 35.47) on day 28.

**Figure 1:**
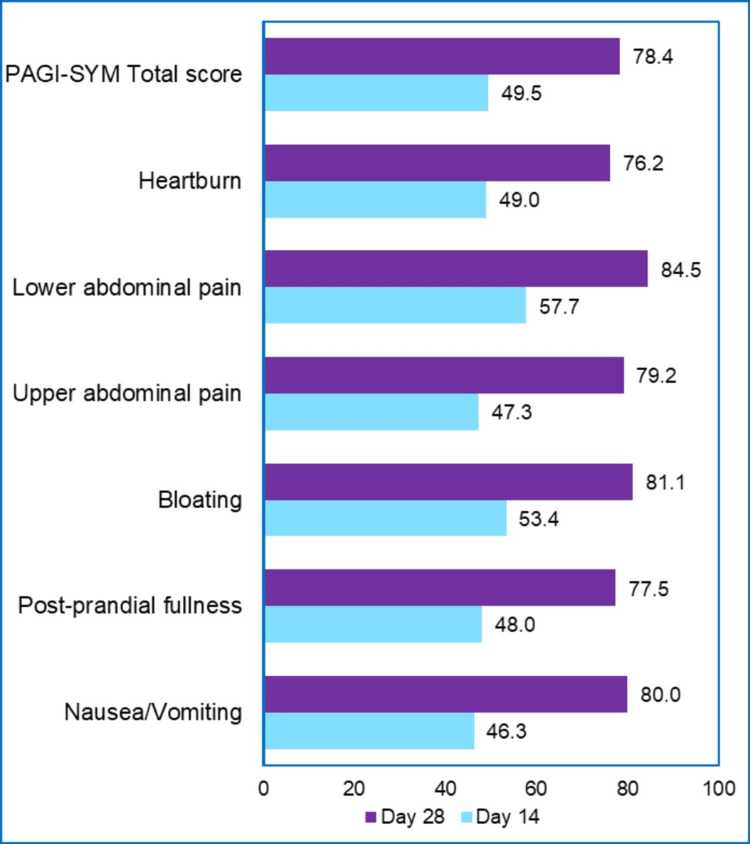
Percent change from baseline in PAGI-SYM (total and domain) scores PAGI-SYM: Patient Assessment of Gastrointestinal Disorder Symptom Severity Index.

Table 2 presents the baseline and post-treatment PAGI-SYM (total and domain) scores. There were significant reductions in the individual symptom scores on day 14 (p < 0.0001). The total PAGI-SYM scores at baseline were 41.32 (15.487), which reduced to 20.86 (11.620) on day 14 (p < 0.0001). Figure 1 presents the percent change in mean PAGI-SYM scores from baseline to days 14 and 28. The total PAGI-SYM scores were reduced by 49.5% on day 14 and 78.4% on day 28 with omeprazole therapy. Similarly, there was a significant reduction in all domain scores for the PAGI-SYM scale.

**Table 2:** PAGI-SYM scores at baseline, day 14, and day 28 * A positive value indicates reduction; ** Wilcoxon test; # p < 0.0001 (Friedman test comparison of baseline versus day 14 and day 28 scores for all domains and total score); IQR: interquartile range; SD: standard deviation; SE: standard error; PAGI-SYM: Patient Assessment of Gastrointestinal Disorder Symptom Severity Index.

					Paired differences from baseline					
	Median	IQR	Mean	SD	Mean*	SD	SE	95% CI	Z score**	p-value
Nausea/vomiting										
Baseline	4.00	5.00	4.36	2.821						
Day 14	2.00	3.00	2.34	1.758	2.02	2.32	0.24	1.55 to 2.49	6.90	<0.0001
Day 28	0.00	1.50	0.88	1.207	3.49	2.75	0.28	2.93 to 4.05	7.74	<0.0001
Post-prandial fullness										
Baseline	8.00	5.50	8.50	3.713						
Day 14	4.00	5.00	4.42	2.940	4.08	2.97	0.30	3.48 to 4.69	8.26	<0.0001
Day 28	1.00	3.00	1.92	2.306	6.58	3.51	0.36	5.87 to 7.29	8.46	<0.0001
Bloating										
Baseline	4.00	4.00	3.86	2.227						
Day 14	1.00	2.50	1.80	1.600	2.06	1.70	0.17	1.72 to 2.41	7.85	<0.0001
Day 28	0.00	1.00	0.73	1.041	3.14	2.06	0.21	2.72 to 3.55	8.04	<0.0001
Upper abdominal pain										
Baseline	5.00	2.00	4.71	1.660						
Day 14	2.00	2.50	2.48	1.384	2.23	1.49	0.15	1.93 to 2.53	7.82	<0.0001
Day 28	1.00	1.00	0.98	1.086	3.73	1.73	0.18	3.38 to 4.08	8.27	<0.0001
Lower abdominal pain										
Baseline	4.00	3.00	3.57	2.071						
Day 14	1.00	3.00	1.51	1.589	2.06	1.81	0.18	1.70 to 2.43	7.33	<0.0001
Day 28	0.00	1.00	0.55	0.905	3.02	1.97	0.20	2.62 to 3.42	8.10	0.001
Heartburn										
Baseline	17.00	10.00	16.31	7.052						
Day 14	8.00	7.00	8.31	5.210	8.00	6.46	0.66	6.69 to 9.31	8.07	<0.0001
Day 28	3.00	4.00	3.88	3.599	12.44	6.98	0.71	11.02 to 13.85	8.36	<0.0001
PAGI-SYM total score										
Baseline	41.00	25.50	41.32	15.487						
Day 14	19.50	17.00	20.86	11.620	20.46	13.87	1.42	17.65 to 23.27	8.50	<0.0001
Day 28	6.00	9.50	8.93	8.361	32.40	15.19	1.55	29.32 to 35.47	8.48	<0.0001

TSQM scores

Table 3 presents the TSQM (total and domain) scores on days 14 and 28. The scores for TSQM were higher on day 28 than on day 14 for all domains of effectiveness, convenience, and global satisfaction. Also, the total TSQM scores were higher on day 28 from day 14, which suggests that omeprazole therapy improves treatment satisfaction over a period.

**Table 3:** Patients' overall treatment satisfaction (TSQM) scores on days 14 and 28 SD: standard deviation; TSQM: Treatment Satisfaction Questionnaire for Medication.

	N	Mean	SD	Median	Min.	Max.	Range
TSQM domain: day 14							
Effectiveness	96	23.34	17.19	15.00	4	58	54
Convenience	96	13.05	5.87	15.00	3	21	18
Global satisfaction	96	9.78	4.18	11.00	3	17	14
Total TSQM score	96	34.69	14.03	40.00	12	58	46
TSQM domain: day 28							
Effectiveness	96	25.28	17.75	20.50	5	63	58
Convenience	96	12.82	6.01	15.00	3	21	18
Global satisfaction	96	10.43	5.09	12.00	2	21	19
Total TSQM score	96	36.67	15.97	41.50	13	63	50

Reflux

Reflux symptoms were reported by 92 (95.83%) patients at baseline, whereas only 29 (30.21%) patients reported reflux on day 28. Thus, there was significant (p < 0.0001) improvement seen in 46.74% (43/92) of patients on day 14 and 68.48% (63/92) of patients on day 28. Clinically significant findings were observed in 37 (38.54%) patients at baseline. Oral omeprazole therapy reduced the number of patients with clinically significant findings to only 16 (16.67%) patients on day 14 and eight (8.33%) patients on day 28.

Safety

A total of four (4.17%) patients reported four treatment-emergent adverse events, which were abdominal pain, headache, constipation, and diarrhea. All events were of mild severity and were reported as unrelated to the study medication by the treating clinician. Dose modification was required only for one patient who reported constipation. All events were resolved without sequelae, and there were no serious adverse events.

## Discussion

The findings of this real-world evidence study show the effectiveness of omeprazole in the treatment of patients with APD (duodenal ulcer, gastric ulcer, reflux oesophagitis) based on the self-reported outcomes by patients. These observations are consistent with the existing literature and evidence, which suggests that omeprazole is safe, effective, and well-tolerated in APD. In a study by Lazebnik LB et al. (2021), omeprazole provided greater improvement in dyspepsia assessed using the Short-Form Leeds Dyspepsia Questionnaire (SF-LDQ) in uninvestigated heartburn in patients with heartburn (GERD and gastritis; n = 10,509) [10]. In our study, the total PAGI-SYM scores improved significantly (p<0.0001) from 41.32 (15.487) at baseline to 20.86 (11.620) on day 14, and 9.27 (2.07) on day 28. In an analysis of a Danish multicenter trial, a high BMI, the use of antacids or H2-blockers within the last month, or pain at night-time were independently associated with good omeprazole response to dyspepsia treatment, whereas the presence of nausea was associated with a poor omeprazole response [11]. We observed significant improvement in the number of patients reporting reflux on day 28 (p = 0.001). In a factor analysis of data from double-blind, randomized, placebo-controlled trials (the Bond and Opera studies), Talley NJ et al. (1998) concluded that TSQM is a psychometrically sound and valid measure of the major dimensions of patients' satisfaction in functional dyspepsia [12]. We used the TSQM scale to assess treatment satisfaction and observed that there were significant improvements (increase) with omeprazole therapy. The total TSQM mean (SD) scores on day 14 and day 28 were 34.69 (14.03) and 36.67 (15.97), respectively.

PPIs are preferred for acid-related diseases like APD, including GERD, peptic ulcer disease (PUD), and ZES [13]. They improve the symptoms of dyspepsia and APD, improve gastric myoelectrical activity, and do not affect gastric motility [14]. They suppress the growth of *Helicobacter pylori*, and when administered in combination with antimicrobial agents, provide the best treatment option for eradication of the bacterium. PPIs administered once daily showed endoscopic evidence of healing in >90% of patients with duodenal ulcers and gastric ulcers after four weeks and six weeks of treatment, respectively [13]. Eight-week PPI therapy also has been reported to improve endoscopically proven healing in patients with ulcerative or erosive GERD [13]. The authors further state that daily doses of a PPI are effective in preventing relapse of GERD. As reported by Zimmermann AE et al. (2001), omeprazole has been administered to children aged two months to 18 years for the treatment of erosive esophagitis, gastric ulcer, duodenal ulcer, *Helicobacter pylori* infection, and related conditions at dosages of 5-80 mg/d (0.2-3.5 mg/kg/d) for periods ranging from 14 days to 36 months with a low incidence of adverse effects [15]. The initial dose most consistently reported to heal esophagitis and provide relief of symptoms of GERD appears to be 1 mg/kg per day. The authors conclude that in uncontrolled clinical trials and case reports to date, omeprazole is effective and well tolerated for the acute and chronic treatment of esophageal and PUD in children, particularly those who had failed to respond to previous treatment with H2-receptor antagonists.

The AEs of special interest were gastrointestinal intolerance, abdominal pain, diarrhea, nausea, vomiting, heartburn, giddiness, and regurgitations. Out of 96 patients, only four patients reported adverse events (constipation, abdominal pain, headache, and diarrhea). All events were mild in severity and omeprazole therapy was well tolerated. While omeprazole is safe and well tolerated in all short-term studies, uncertainty persists with respect to the long-term consequences of hypergastrinemia caused by profound acid suppression, and additional studies are needed to address this concern [16,17].

Recommendation of the study

This study being a real-world evidence study, the findings relate to the patient population at large, and hence the results of this study can be generalized to patients with APD. A larger study sample could have offered a sub-group analysis based on various factors like etiology, gender, dietary habits, lifestyle, and use of other drugs.

Limitations of the study

Our study has limitations in terms of small sample size, shorter four weeks of study duration, and lack of comparator. Another limitation of the study is that no objective assessment of APD was conducted. Although PRO scales were used, they can be influenced by patient bias and patient-level response behaviors. However, the main strength of this study is the outcomes as reported by patients in real-world settings.

## Conclusions

In conclusion, this real-world clinical study assessed the outcomes of four-week omeprazole 20/40 mg per day therapy in patients with APD. Omeprazole has been widely used to prevent and manage several acid-related disorders. Omeprazole provides significant improvement in PAGI-SYM and TSQM scores on day 14 and day 28. Patients reported the omeprazole-based therapy as effective, convenient, and satisfactory. Omeprazole therapy is safe and effective for the treatment of APD and shows good improvement in APD in patients suffering from duodenal ulcers, gastric ulcers, and reflux oesophagitis.
